# Perspective—The Feasibility of Continuous Protein Monitoring in Interstitial Fluid

**DOI:** 10.1149/2754-2726/accd7e

**Published:** 2023-04-28

**Authors:** Thomas Young, Vincent Clark, Netzahualcóyotl Arroyo-Currás, Jason Heikenfeld

**Affiliations:** 1 Department of Biomedical Engineering, University of Cincinnati, Cincinnati, OH 45221, United States of America; 2 Chemistry-Biology Interface Program, Zanvyl Krieger School of Arts & Sciences, Johns Hopkins University, Baltimore, MD 21218, United States of America; 3 Department of Electrical & Computer Engineering, University of Cincinnati, Cincinnati, OH 45221, United States of America; 4 Department of Pharmacology and Molecular Sciences, Johns Hopkins University School of Medicine, Baltimore, MD 21205, United States of America

## Abstract

Real-time continuous monitoring of proteins in-vivo holds great potential for personalized medical applications. Unfortunately, a prominent knowledge gap exists in the fundamental biology regarding protein transfer and correlation between interstitial fluid and blood. Additionally, technological sensing will require affinity-based platforms that cannot be robustly protected in-vivo and will therefore be challenged in sensitivity, longevity, and fouling over multi-day to week timelines. Here we use electrochemical aptamer sensors as a model system to discuss further research necessary to achieve continuous protein sensing.

## Highlights

- Continuous monitoring or sensing of proteins.
- Biological challenges to continuous sensors for proteins.
- Biosensor sensitivity, longevity, and antifouling are areas for future research.

Most diagnostic assays for disease management measure large biomarkers such as proteins. It is, therefore, easy to envision that continuous monitoring of protein biomarkers could be revolutionary for the management of diseases such as heart disease, inflammatory bowel disease, diabetes, etc. Building on the successes seen in continuous glucose monitoring, the ideal protein biomarker is likely one which is present in interstitial fluid (ISF) at detectable concentrations,^1^ correlates closely with physiologically relevant systemic concentrations, and is measurable with adequate accuracy to enable clinically-informed action.

However, the leap from small molecule monitoring to protein monitoring is quite large. There is a fundamental knowledge gap regarding both the biological and technological feasibility of continuous protein sensing. First, with respect to biology, it is yet to be confirmed with significant accuracy how large proteins (similar to or greater than the molecular weight of albumin) partition from blood to dermal ISF, leading to unknown correlations between protein levels in blood vs ISF.^2^ Furthermore, it is unclear how external stimuli from insertion of sensors into the body will affect protein transport between blood and ISF (i.e., inflammatory response). Second, technologically, it is unknown if affinity-based sensing mechanisms such as redox-modified aptamers can accurately measure any type of protein in ISF over the multi-day to multi-week time scale needed for meaningful health status assessments. When deployed in biological fluids, if the sensors are to measure proteins, they cannot be robustly membrane protected like glucose sensors (or the protein targets would be filtered out). Additionally, the sensors will undergo multi-modal degradation based on interactions with small molecules and proteins. Therefore, it has yet to be determined if there is an effective method to protect sensing interfaces while still allowing affinity probes to specifically interact with protein biomarkers.

The present perspective focuses on biological and technological concerns regarding continuous protein monitoring. The focus begins with the biology of protein monitoring and then moves into technological advances because without first establishing biological feasibility, continuous protein monitoring technology is not worth contemplation. Electrochemical aptamer sensors will be used as a model case study when discussing challenges to protein monitoring due to their previous in vivo demonstrations.^3,4^ Even though aptamer sensors will be exclusively discussed, the problems they have are the same as any other affinity based platform which could be used (antibodies, enzymes). Therefore, the presented information can inform future research in all affinity-based platforms.

## Biology

**Figure 1.**
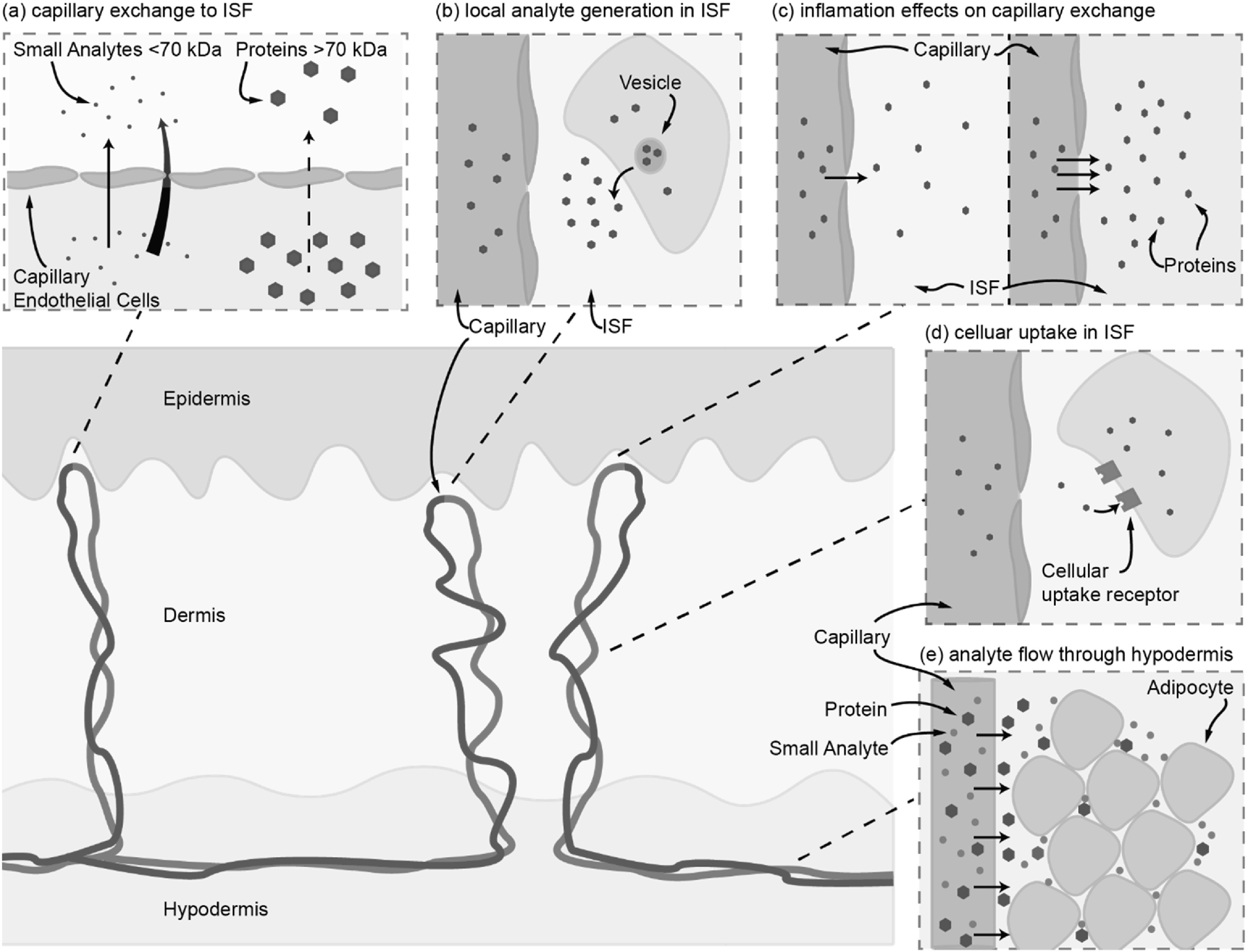
Several challenges related to biology are inherent to continuous protein monitoring. (a) Protein concentration correlations and lag time between blood and ISF could be skewed due to proteins’ large size. (b) Local generation of proteins by cells in ISF could impact concentration correlations with blood. (c) External factors such as inflammation or skin temperature could impact capillary permeability. (d) Cells in ISF could uptake proteins for use. (e) The physical tissue environment based on cellular content could impact protein distribution for detection.

Biosensors have broadly been developed for a wide range of sensing media such as interstitial fluid (ISF),^1^ saliva,^5^ sweat,^6,7^ and blood.^3^ While most of these biofluids can have niche instances of use for specific biomarkers, only blood and its next most similar biofluid ISF have a high potential for measuring proteins with clinical significance. ISF stands out further with potentially the most practical value because of its ease of access for continuous monitoring. ISF is readily available in the dermis and hypodermis of the skin where almost all analytes found in blood can also be found in ISF.^2^ Even though ISF presents an opportunity to continuously monitor proteins, several fundamental challenges (Fig. 1) related to biology present themselves including:

1. How are proteins’ lag times and correlations to blood impacted by capillary filtration from blood into ISF?
2. How does local generation of biomarkers confound concentration correlations with blood?
3. How does the local physical tissue environment impact protein diffusion and transport to the sensors?
4. How does the local physical tissue environment impact protein concentration due to cellular uptake at membrane receptors?
5. How does protein exchange fluctuate based on external factors such as skin temperature, diurnal effects, or drugs taken?

The answers to these challenges will inform the technological design for continuous protein sensors and progress the field forward.

### Lag times/concentration

The first challenge concerns the ability of proteins to pass into ISF, how long the transport takes, and the dilution factor of the protein. Previous research has shown that most analytes (and therefore proteins) found in blood are also found in ISF; however, the size of proteins greatly determines their relative concentrations in ISF.^2^ Proteins smaller than albumin (∼66 kDa) can pass from blood into ISF unassisted through paracellular transport or assisted through transcellular transport. The size filtration of albumin from blood into ISF does cause albumin concentrations in ISF to be about 50% of blood concentrations.^8,9^ Therefore, proteins smaller than albumin see a similar or lesser decrease in relative concentrations in ISF. Proteins larger than the size of albumin do see a large concentration decrease in ISF due to their transcellular transport requirement.^2^ This concentration decrease could be below sensing threshold or highly variable and therefore poorly correlated with blood. Such concentration changes represent fundamentally important and yet unanswered questions.

The size exclusion from capillaries not only impacts ISF concentrations, but also the lag times between protein concentration spikes in blood and equilibration with ISF. Compared to relative analyte concentrations between blood and ISF, lag times for larger analytes have not been well studied.^1^ This presents an opportunity for future research and sensor development because the best way to accurately measure lag times in ISF is through an indwelling sensor in the dermis or hypodermis, which should be more accurate in lag-time measurements than ISF extraction or microdialysis.^2^

### Local generation

One challenge to correlating ISF protein concentrations to blood concentrations is that some proteins are produced locally in dermal ISF, but then circulated in the blood to other organs. Interleukin-6 (IL-6) represents an excellent example of local generation impacting blood correlations. IL-6 is synthesized in the local tissue environment in response to infection or tissue injury where it then enters the blood.^10^ By being locally generated in ISF, the spike in concentration detected by an indwelling sensor could occur prior to the spike in blood, or vice versa if the infection or injury originates elsewhere in the body than the measurement site. Confounding factors related to proteins which are locally generated need to be considered during sensor development and in down-selecting potential clinical use cases.

### Physical tissue environment

The most accessible location for ISF sensing is in the skin which is composed of three layers: epidermis, dermis, and hypodermis.^11^ Each layer contains unique cells and mechanical properties which will impact protein movement and transport throughout. The epidermis contains tightly packed cells connected through tight, gap, and adherens junctions to create an impermeable barrier to large solutes.^12^ The tight connections also contribute to a low volume of ISF available in this layer. The dermis is located directly beneath the epidermis and is a moderately vascularized matrix of collagen with a low cell density.^2^ Therefore, the vascularization and low cellular count of the dermis could make it an ideal location for continuous protein monitoring. The hypodermis contrasts the dermis with a highly dense cellular content of adipose cells and low ISF content;^2^ therefore, it is possible to speculate that the hypodermis could be inferior to the dermis. However, this all depends on the target to be measured, and if the measurement goal is not just blood correlation but rather tissue penetration of a protein. In the latter case, the hypodermis could be the preferred measurement location.

### Local protein capture or modification

After arrival into ISF and dispersion throughout, many proteins are chemically altered or captured by receptors on cells for use and signaling. As a beautiful example, a study conducted by Yang and colleagues compared two similar proteins, inulin and insulin, and each protein’s transport from blood into ISF and lymph. Their results showed that although inulin reached an equilibrated concentration between ISF, plasma, and lymph, insulin did not reach the same equilibration. Instead, insulin concentrations were consistently lower in ISF and lymph as compared to plasma, showing insulin’s ability for uptake into cells.^13^ A similar situation occurs with brain natriuretic peptide (BNP) and adipose tissue where adipocytes have numerous receptors on their surface for BNP clearance.^14^ These results highlight the fact that protein transport and equilibration into ISF is a dynamic process with a multitude of factors which can impact protein concentrations in ISF and correlations between ISF and plasma.

### Fluctuate due to external factors

Another challenge related to continuous protein monitoring concerns how factors external to fundamental capillary exchange can impact concentrations in ISF. How do factors such as skin temperature, inflammation, blood pressure, diurnal changes, and pharmaceutical drugs impact capillary exchange and therefore protein concentrations in ISF? Each factor may impact protein concentrations differently and needs to be considered both independently and collectively when interpreting protein correlations in ISF. Temperature, inflammation, and blood pressure are directly related with vasodilation/vasoconstriction and can therefore impact skin blood flow, and in the case of inflammation also modulate vascular permeability.^15^ Additionally, many protein concentrations change throughout the day. For example, C-reactive protein has been shown to maintain stable concentrations in healthy individuals diurnally,^16^ whereas insulin varies based on time of day and circadian rhythm.^17^ Pharmaceutical drugs can have a broad array of effects on protein concentrations from impacting vascular perfusion and capillary exchange to modulating specific protein production. These are just a few factors which can ultimately have a large impact on a sensor’s ability to correlate relative concentrations in ISF with blood.

We believe two factors will be the greatest obstacles to feasibility: variable dilution during capillary filtration and cellular uptake in the sensing environment. Determining unknown dilution factors will significantly add to the research required because correlations between blood and ISF will need to be determined individually. Additionally, protein uptake by cells impacts concentrations available for a continuous sensor to detect. Overall, we believe the greatest biological challenge will be one which variably reduces concentrations available for sensing.

A final question, at least in the limited breadth of this perspective piece, concerning biological challenges for continuous protein monitoring in ISF is what if the summation of the described obstacles above cannot be overcome with technology? The next best biofluid containing proteins of interest would be whole blood, so could a sensor be placed in the bloodstream? Continuous sensing in blood solves the main problems of correlating concentrations and equilibration lag times but presents several new challenges concerning technological feasibility of a blood-based sensor. Sensor design would become a higher concern in blood to not disrupt blood flow, elicit an immune response, induce clotting, introduce infectious pathogens, or maintain stable positioning. Additionally, comfort and ease of use could be a concern in a commercialized product for patients. This being said, commercial catheters for patients who need regular dialysis exist. However, while this question currently does not have one answer, it should still be considered as a potential option if technology cannot accommodate the requirements for ISF sensing.

## Technology

Current sensing modalities are dominantly based on one of two fundamental mechanisms: affinity-based and reactivity-based interactions. Proteins will in almost all cases require affinity-based sensors because even if an enzyme could be developed that would react with a protein and create a detectable byproduct, the concentrations of proteins are typically so low and their diffusion rates to such a sensor would be so slow that limit of detection could become an insurmountable challenge. The most widely in-vivo demonstrated class of affinity biosensor is aptamer sensors, and more specifically, only redox-tagged aptamer sensors being shown to work in-vivo (impedimetric and other approaches are more challenging in whole biofluid). Despite compelling initial demonstrations,^3,4^ current aptamer sensors suffer from sensitivity, longevity, and fouling concerns inhibiting their progression towards continuous protein sensing. Aptamer sensors will therefore be used as a model case study to discuss continuous in vivo protein monitoring, but each challenge persists across all affinity-based biosensors.

## Sensitivity and Limit of Detection

### Current status

Aptamers selected for proteins via the SELEX process can often have very low limits of detection due to increased binding affinity (Kd) between aptamer and protein. Simply, proteins are larger and more structurally and chemically complex than small molecules, allowing a greater degree of binding interactions with an aptamer. Commercially, companies such as Somalogic have further advanced binding affinity through chemically modified aptamers against >6000 proteins.^18^ For example, Somalogic has an aptamer for IL-6 with a binding affinity of 200 pM.^19^

### Future needs

Although binding affinities for proteins appear promising,^20,21^ sensitivity remains a bigger challenge for protein aptamer sensors. Unlike small molecule aptamers which show changes in redox tag current (sensitivity) over 100’s of percent throughout the detection range, aptamer sensors for proteins have been more limited with typical ranges of 10’s of percent. A portion of the challenge is that for small molecules many aptamers bind and form a linked stem geometry resulting in a strong redox current change between the electrode and redox-tagged aptamer. Proteins are often so large and have many aptamer-protein binding interactions that a powerful stem-loop or similar aptamer sensor architectures are typically not found in as-selected aptamers for proteins.^20–22^ Thus, further research is needed to optimize such aptamers for high sensitivity protein monitoring.

## Monolayer Longevity

### Current status

Unlike most small molecule analyte targets, most proteins of interest change not in minutes but over hours or days; therefore, in practice, proteins require a sensor capable of operating days as opposed to hours to detect diagnostically relevant information.^23^ Historically, aptamer sensor monolayers on gold electrodes have remained stable in simple buffers like PBS for 48 h under continuous voltammetric interrogation,^24^ but once deployed in vivo, sensors often degrade quickly over time spans of 6–12 h.^3^ Recent work has been performed to identify the driving mechanisms of degradation. Leung et al. reported that electrochemically driven desorption, and fouling of blood components are the primary sources of sensor degradation.^25^ Clark and Pellitero recently showed this degradation is a multi-modal process consisting of voltage induced desorption at low voltages (<−0.2 V), and competitive displacement from thiolated small molecules (i.e. L-cysteine) commonly found in biofluids.^26^ Scanning sensors at negative potentials is one major source of sensor degradation caused by: the reduction of oxygen into reactive oxygen species, thereby catalyzing the loss of monolayer elements from the sensor surface,^27^ the phosphate backbone of DNA being strained away from the electrode surface causing electrochemical desorption of aptamer,^25^ and increased fouling from attracted proteins.^28^ Researchers recently reported no loss in DNA signal following continuous interrogation in the voltage window of −0.2 V to 0.2 V vs Ag|AgCl.^26^ While promising, this voltage range is outside the reduction potential of methylene blue, the benchmark redox tag. Recent work in the field led to the development of novel redox tags including, a tetrathiafulvalene based reporter (E°’ = 0.05 V vs Ag|AgCl),^29^ and an osmium-based redox reporter(E°’ = 0.18 V vs Ag|AgCl).^30^

### Future needs

While these results are promising, further work must be done to progress the field towards longer lasting gold electrode based sensors. A remaining issue is the inherent thermal stability of the monolayer of aptamers and protective molecules such as alkythiolates. Recent work from Watkins and Karajic showed that continuous voltametric scanning along with increasing alkyl chain length from the benchmark 6-mercapto-1-hexanol (MCH) to 8-mercapto-1-octanol (MCO) increases stability in biofluid conditions for over one week.^31^ However, while sensors were able to stay intact, sensor responsivity suffered unless a protective membrane was added to prevent fouling, a sensor membrane so tight it would preclude protein monitoring, setting up the next topic of antifouling.

## Antifouling

### Current status

**Figure 2.**
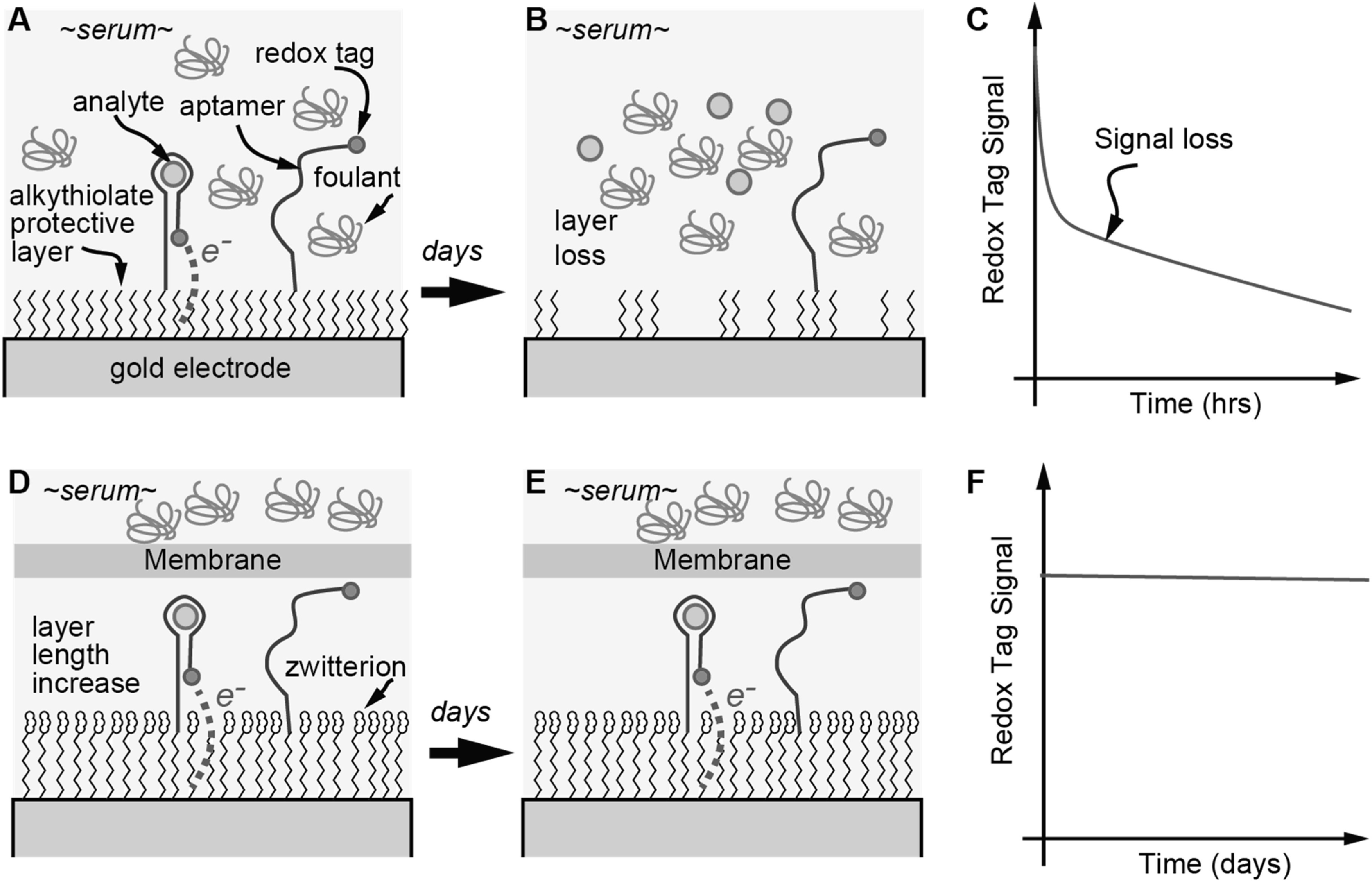
Sensor fouling deployed in biofluids. (A) Aptamer sensors rely on mixed self-assembled monolayers with bonds between a gold electrode and alkythiolates serving as a blocking layer and an alkythiolate modified aptamer with a terminal redox reporter. In the presence of analyte, the aptamer folds bringing the redox tag closer to the sensor surface. When deployed in serum, foulants interact with sensor architecture. (B) Following continuous interrogation, there is extensive monolayer and aptamer loss leading to (C) loss of redox tag signal over the timeline of hours. (D) The addition of a protective membrane and/or an antifouling zwitterion monolayer protects sensor interfaces against unwanted fouling. (E) Upon continuous interrogation, redox tag signal is retained.

Exposing an aptamer sensor to proteins is not necessarily a concern in terms of enzymatic attack of the sensor because aptamers can also be designed to be resistant to nucleases.^32^ A more challenging task is preventing fouling, which is a bigger challenge for protein monitoring because to monitor the protein, you must also allow common foulants such as albumin to reach the sensor surface (Fig. 2). Foulants can also accelerate alkythiolate monolayer desorption^31^ further reducing the longevity of the sensor.

### Future needs

The mechanisms by which a surface fouls have been extensively studied, finding that the best antifouling surface modifications are hydrophilic, hydrogen bond donors/acceptors, and electrically neutral.^33^ All of these properties contribute to highly hydrated surfaces which bind water tightly, creating an effective “water barrier” against foulants to reach the sensor surface. Packing density of surface molecules also contributes to surface hydration by increasing available hydrogen bonds.^34^ Considering all of the ideal characteristics of an antifouling layer, one class of molecules stands out with strong promise for affinity-based protein sensors: zwitterionic molecules.

Zwitterions are charged, extremely hydrophilic species allowing coated surfaces to hydrate readily. These properties could make them an ideal antifouling layer, but little research has been done on their use with affinity-based sensors. In a demonstration of a zwitterionic phosphatidylcholine terminated molecule by Li and colleagues, the aptamer sensors protected by this zwitterion were able to maintain no evidence of fouling for 12 h in whole blood.^35^ Furthermore, as demonstrated by Watkins up to 3 days of antifouling operation was achieved.^31^ However, zwitterionic chemistry is often added to a monolayer with a cost, such as sacrificing monolayer packing density, and/or creating a monolayer that is so thick it impedes redox transfer. Therefore, further research is needed to demonstrate robust antifouling aptamer sensors that remain sensitive even without membrane protection.

We hypothesize an ideal protein biosensor would contain a zwitterionic monolayer to aid in biocompatibility and reduce fouling at the sensor interface with a size exclusion membrane to selectively differentiate between proteins of interest, and non-specific foulant interactions. To combat the muted signal gain obtained during protein sensing two things may be helpful: (1) introducing chemically-modified DNA^36^ that is optimized-post SELEX for an optimal shape-conformation change upon binding to the protein; (2) consideration of optical-based aptamer approaches which can alleviate the need to have the aptamer so immediately close to the substrate it is bound to and allow improved interaction with the protein (waveguide, aptamer bound in a hydrogel, etc.).

## Conclusions

Affinity based sensors provide platforms capable of continuous protein monitoring to advance the field of personalized medicine. However, before technological advances can be made, fundamental questions in biology must be addressed which determine available concentrations and correlative value for detection. Once biological concerns are addressed, increasing sensor longevity and inhibiting fouling in biofluids without the use of membranes will be crucial for long-term sensing. Furthermore, maintaining high sensitivity of the recognition element in complex environments will be necessary to obtain accurate multi-day measurements. Nevertheless, continuous protein monitoring has the potential to revolutionize personalized medicine, changing the lives of millions suffering from chronic diseases all over the world.
